# The role of Nrf2 in astragaloside IV-mediated antioxidative protection on heart failure

**DOI:** 10.1080/13880209.2020.1849319

**Published:** 2020-11-30

**Authors:** Yan-Bo Sui, Kui-Kui Zhang, Yu-kun Ren, Li Liu, Yan Liu

**Affiliations:** a Department of Cardiology, First Affiliated Hospital of Heilongjiang University of Chinese Medicine, Harbin, China; b Department of Dermatology, First Affiliated Hospital of Heilongjiang University of Chinese Medicine, Harbin, China;; c Department of Scientific Research Management, First Affiliated Hospital of Heilongjiang University of Chinese Medicine, Harbin, China

**Keywords:** Oxidative stress, HO-1, Keap-1, transcriptional activation

## Abstract

**Context:**

Heart failure is one of the most serious diseases worldwide. Astragaloside IV (ASI) is widely used in the treatment of cardiovascular diseases.

**Objective:**

To elucidate the antioxidative mechanism of ASI in a rat model of left coronary artery ligation.

**Materials and methods:**

Left coronary artery of Sprague–Dawley rats was ligated to establish the model of heart failure, and then vehicle (saline) or ASI (1 mg/kg/day) was orally administered to the rats (*n* = 15) for 6 weeks. Echocardiography was used to evaluate the cardiac function. Myocardial infarct size was measured by triphenyltetrazolium chloride staining. Oxidative stress in the ventricular myocardium was determined. Molecular mechanisms were investigated by Western blot and chromatin immunoprecipitation.

**Results:**

ASI improved the cardiac function, especially ejection fraction (75.27 ± 5.75% vs. 36.26 ± 4.14%) and fractional shortening (45.39 ± 3.66% vs. 17.88 ± 1.32%), and reduced the infarct size of left ventricle (20.69 ± 2.98% vs. 39.11 ± 3.97%). ASI maintained the levels of glutathione, catalase and superoxide dismutase and prevented the leakage of creatine kinase. In addition, ASI induced the protein expression of Nrf2 (1.97-fold) and HO-1 (2.79-fold), while reduced that of Keap-1 (0.77-fold) in the ventricular myocardium. In H9c2 cells, a rat cardiomyocyte cell line, ASI induced the translocation of Nrf2 from cytoplasm to nucleus, followed by transcriptional activation of *NQO-1* (8.27-fold), *SOD-2* (3.27-fold) and *Txn-1* (9.83-fold) genes.

**Discussion and conclusions:**

ASI prevented heart failure by counteracting oxidative stress through the Nrf2/HO-1 pathway. Application in clinical practice warrants further investigation.

## Introduction

Heart failure, sometimes known as congestive heart failure, occurs when the heart fails to pump blood sufficiently to maintain blood flow to meet the needs of the body (Kenchaiah et al. 2002). With approximately 40 million people worldwide being affected annually, heart failure becomes the most serious health problem in developed and developing countries (Savarese and Lund 2017). A number of factors, such as ischaemic heart disease, hypertension, dyslipidemia, diabetes, and smoking, are reported to increase the risk of heart failure (Bui et al. 2011).

It is well known that the heart is susceptible to oxidative stress, which is defined as the excessive production of reactive oxygen species (ROS) relative to antioxidant defense (Chen et al. 2018). Oxidative stress plays an important role in the pathophysiological processes of cardiac remodelling and heart failure. Excessive ROS can cause cell dysfunction, protein and lipid peroxidation, DNA damage, irreversible cell damage, and death, all of which are related to a range of pathological cardiovascular diseases (Tsutsui et al. 2008).

Nuclear-factor-E2-related factor (Nrf)-2 is a member of bZIP transcription factors, which are expressed in a variety of tissues (Nguyen et al. 2009). Most of the antioxidants and detoxifying enzyme genes contain specific nucleotide sequences, such as antioxidant response element (ARE), in their promoters. Transcriptional activation of the antioxidant genes by ARE is largely dependent upon the activation of Nrf2 (Lee and Johnson 2004). The genes that contain a functional ARE include those encoding haem oxygenase-1 (HO-1), superoxide dismutase (SOD), glutathione peroxidase (GPx), glutathione-*S*-transferases (GST), NAD(P)H:quinone oxidoreductase-1 (NQO-1), NQO-2, thioredoxin-1 (Txn-1), and thioredoxin reductase-1 (Txnrd-1), all of which play a role in protecting against oxidative stress (Li et al. 2010).

*Astragalus propinquus* Schischkin (Fabaceae) has been used to treat various diseases for thousands of years (Li et al. 2017). Astragaloside IV (ASI), as the main active component of the plant extract, has therapeutic potential against cerebral ischaemia/reperfusion (Yuan et al. 2018), cardiovascular disease (Tang et al. 2018), pulmonary disease (Qian et al. 2018), liver fibrosis (Zhao et al. 2020), and diabetic nephropathy (Liu et al. 2017). ASI has been shown to improve cardiac function and inhibit compensatory hypertrophy and ventricle remodelling in rats with congestive heart failure (Wang et al. 2010; Tang et al. 2018). Our previous study reported that ASI alleviated heart failure by promoting angiogenesis through a JAK–STAT3 pathway (Sui et al. 2019). However, it is not clear whether the effect of ASI on heart failure is related to the mechanism of antioxidation. The present study elucidates the mechanism of ASI in a rat model of left coronary artery ligation.

## Materials and methods

### Animals

Adult male Sprague–Dawley (SD) rats (200–220 g) were purchased from Vital River (Beijing, China) and preserved in standard laboratory conditions (temperature 26 °C, humidity 52–60%). All animal care and experimental programs conformed to the animal management regulations of China Food and Drug Administration and were approved by the Animal Care and Use Committee of Heilongjiang University of Traditional Chinese Medicine.

### Drug

ASI of over 98% purity was purchased from Sigma Aldrich (St. Louis, MO). ASI was dissolved in 20% dimethyl sulfoxide and diluted with saline for animal research.

### Model induction and treatment

SD rats, anaesthetized with pentobarbital sodium (30 mg/kg), were connected with an electrocardiograph (ECG) recorder. Surgery to ligate the left coronary artery was performed on 45 rats and for sham ligation on 10 rats. As described previously, permanent ligation of the left coronary artery induced a heart failure model (Fletcher et al. 1981). In brief, the third rib was cut off, the left anterior descending branch of the artery was ligated with 6.0 threads at 2–3 mm near its starting point. The colour change of the ischaemic area (anterior ventricular wall and apex) and the occurrence of arrhythmia (ST-segment elevation) were identified as a confirmation of successful ligation. Within 24 h, 67% of the rats survived the surgery. Animals in the sham control group underwent thoracotomy with pericardium opened but the ligation around the left anterior descending branch was not performed. Subsequent to the surgery (24 h later), the surviving rats were randomly divided into three groups: (1) sham (*n* = 10); (2) heart failure (HF) (*n* = 15); (3) HF + ASI (1 mg/kg/day) (*n* = 15). Animals were given vehicle (saline) or ASI orally for 6 weeks. The weight of rats was measured every 2 or 3 days during the treatment, and the dosage of the drug was adjusted according to the body weight. Animal deaths were recorded every day since the beginning of the treatment.

### Echocardiography

Six weeks after the surgery, rats were anaesthetized with pentobarbital sodium (30 mg/kg) and placed on a heating pad. Vevo770 (Visual Supersonic Company, Toronto, Canada) with a 716 probe was used to dynamically evaluate the cardiac function of the rats. The ventricular structure sensor with a frequency of 17.5 MHz provided spatial resolution. Left ventricular systolic internal diameter (LVIDs) and left ventricular diastolic internal diameter (LVIDd) were measured by M-mode tracings. Ejection fraction (EF) and fractional shortening (FS) were calculated automatically by a high-resolution electrocardiogram system.

### Triphenyltetrazolium chloride staining

After echocardiography, the animals were sacrificed, and their hearts were removed, immediately washed with PBS and frozen and preserved at −80 °C. Six to eight cross-sections of each heart were taken manually. After being soaked in 1% triphenyltetrazolium chloride (TTC) solution at 37 °C for 30 min, the sections were rinsed with saline and fixed in 4% polyformaldehyde for 30 min. Next, the slices were placed on a glass slide, photographed with a digital camera, and the images were analyzed with ImageJ software (NIH, Boston, MA).

### Oxidative stress analysis

The levels of creatine kinase (CK), glutathione (GSH), catalase (CAT) and superoxide dismutase (SOD) in the border zone of infarcted ventricular myocardium were determined with commercially available kits according to the manufacturer’s instructions (Jiancheng Institute of Bioengineering, Nanjing, China).

### Western blot

The ventricular tissue was homogenized in ice-cold lysis buffer using a rotor-stator homogenizer. The proteins were denatured by being mixed with loading buffer (Beyotime, Shanghai, China) and heated at 70 °C for 10 min. This was followed by separating the proteins on an SDS-PAGE gel and transferring the proteins to PVDF membranes. The membranes were blocked with non-fat milk and incubated overnight with primary antibodies (Cell Signalling, Denver, MA) at 4 °C for 24 h. Afterwards, the membranes were incubated with an HRP-conjugated secondary antibody (Cell Signalling) for 1 h. Immobilon solution (Millipore, Billerica, MA) was added to the membrane to develop signals, which were subsequently captured by Fluorchem Image System (Alpha Innotech, Santa Clara, CA).

### Protein extraction and nuclear isolation

H9c2 cells (American Type Culture Collection, Rockville, MD), a rat cardiomyocyte cell line, at 80–90% confluence, were cultured in serum-free DMEM (Invitrogen, Carlsbad, CA) for 24 h, and then treated with vehicle or ASI (50 μg/mL) for 12 h. NE-PER Nuclear and Cytoplasmic Extraction Reagent (Pierce, Rockford, IL) were used to extract the nuclear and cytoplasmic protein according to the manufacturer’s instructions to carry out Western blot and Chromatin immunoprecipitation (ChIP).

### Chromatin immunoprecipitation

ChIP was performed using an Agarose ChIP kit (Pierce) according to the manufacturer’s instructions. ChIP-level antibodies against Nrf2 were obtained from Cell Signalling. Immunoprecipitated DNA was purified by DNA purification column (Tiangen, Beijing, China) and quantified using real-time PCR. The reaction system included 2 μL of cDNA, 12.5 μL of 2 × SYBR Green 1 Master Mix (Fermentas, Vilnius, Lithuania), and 1 μL of each promoter primer. NQO-1 (Approx. −5449 to −5440) 5′ primer: CAAACACTGCCAACCTG. 3′ primer: AGCTAAATCCCCAACCCCTGTGT. SOD-2 (Approx. −3723 to −3714) 5′ primer: CTGAGGGCAAGAGAAAAGAGAT. 3′ primer: TACCCCTAAGTGAGTCCATTGAT. Txn-1 (Approx. −3769 to −3760) 5′ primer: GACAAGGCAAATCACAGAC. 3′ primer: ACACATACACATACATACATCC (Li et al. 2010). PCR was performed using iCycler iQ5 (Bio-Rad, Hercules, CA) at an annealing/extension temperature of 62 °C.

### Statistical analysis

All values were presented as mean ± standard deviation (SD). The one-way analysis of variance (ANOVA) was used to examine statistical comparisons among groups. Two-tailed Student’s *t*-test was used to determine the statistical significance of differences between the two groups. Kaplan–Meier survival curves were compared using a log-rank test. A difference with the *p*-value of less than 0.05 was considered to be statistically significant.

## Results

### ASI improved body weight and survival in rats with heart failure

**Figure 1.**
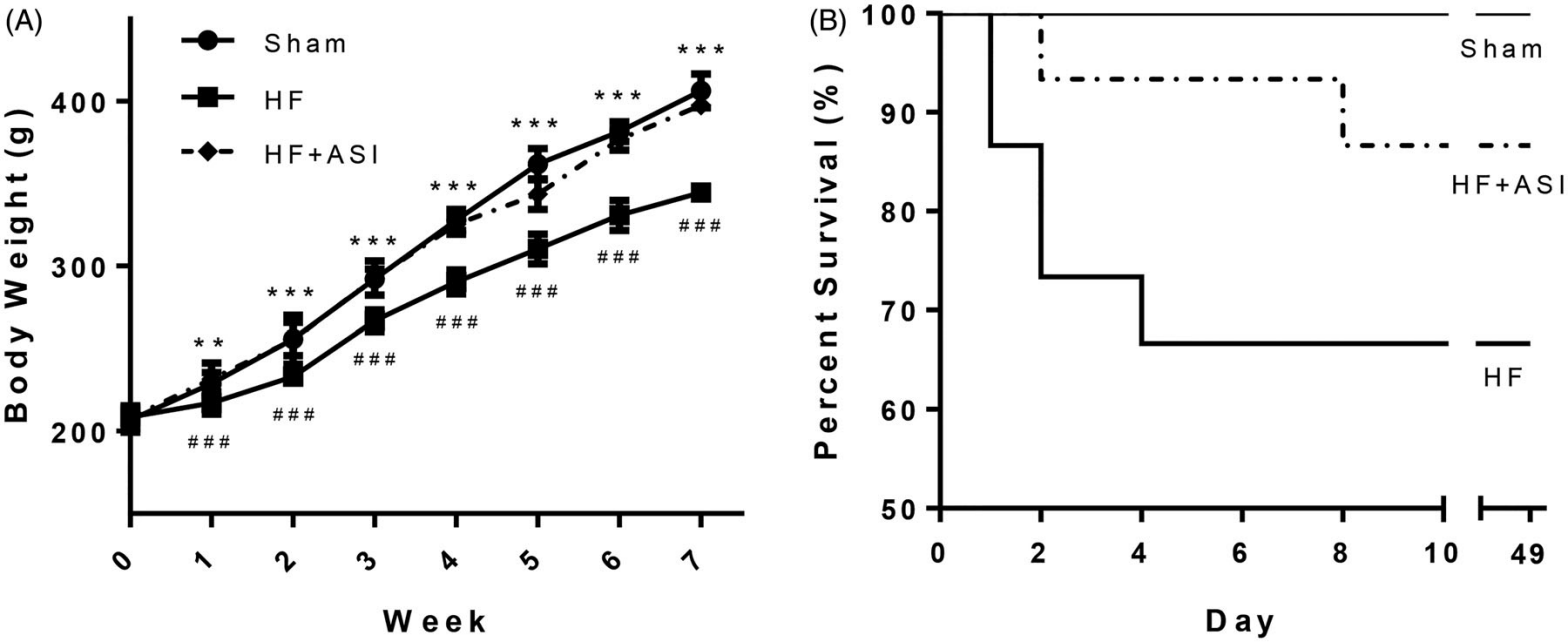
Body weight and survival rate of the rats. Heart failure was induced by ligating the left coronary artery, and rats were orally administered saline (vehicle) or ASI (1 mg/kg/day) for 6 weeks. (A) Body weight. (B) Kaplan–Meier survival curve. Data are presented as mean ± standard deviation (SD). *n* = 10–13. ^###^*p* < 0.001 vs. sham group. ***p < 0.001 vs. HF group. **p < 0.01 vs. HF group.

Left coronary artery ligation resulted in significant weight loss (Figure 1(A)). ASI significantly increased the bodyweight of HF rats (*p* < 0.001 compared with the HF group). In addition, compared with the low survival rate of rats in the HF group (10/15), ASI increased the survival rate of HF rats (13/15), though the improvement in the survival rate was not significant (*p* > 0.05; Figure 1(B)). Body weight and survival data suggested that ASI might be a potential drug that would not cause substantial toxicity in experimental animals.

### ASI improved cardiac function in rats with heart failure

**Figure 2.**
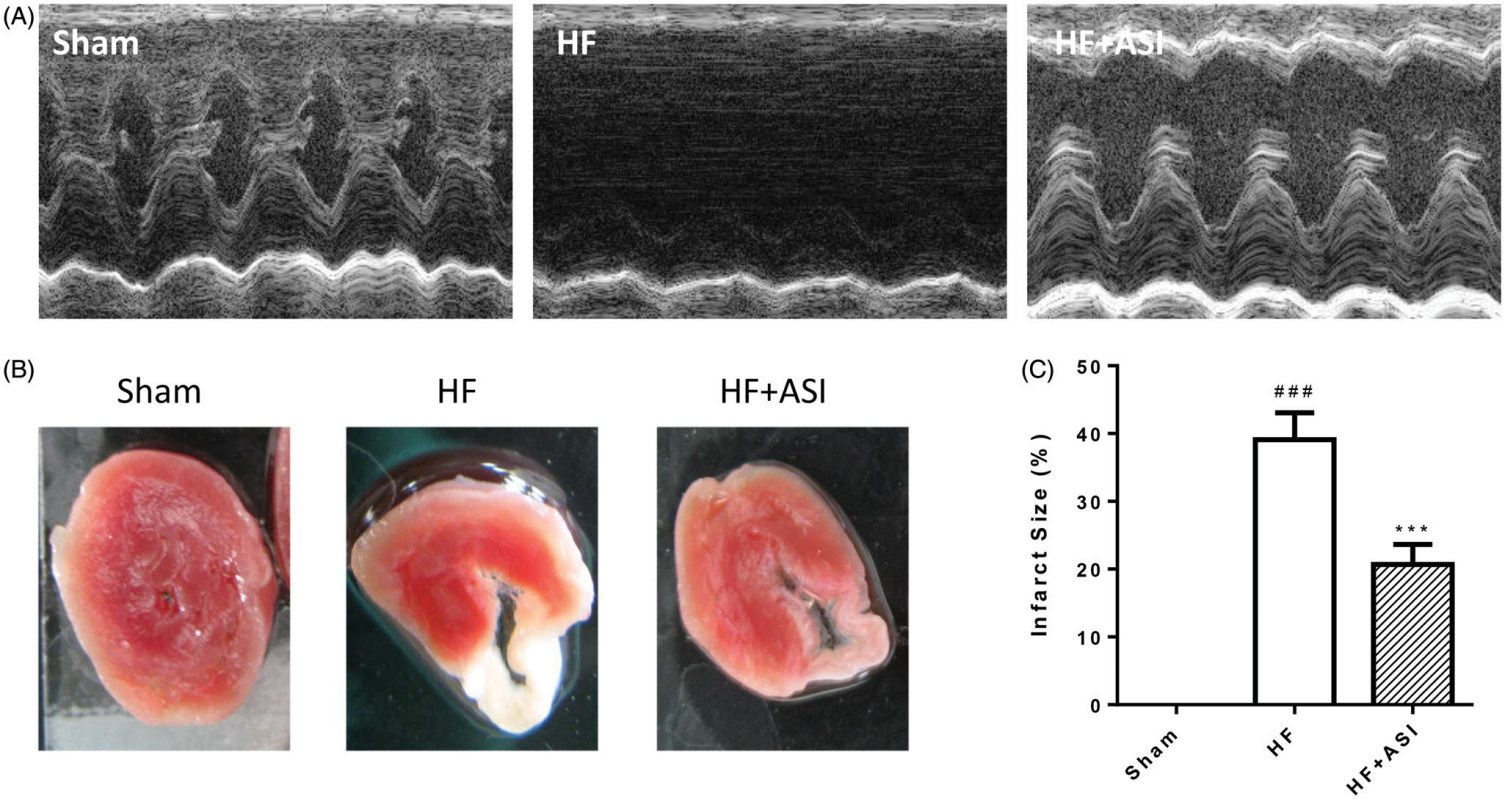
Cardiac function and infarct size of the hearts. Cardiac function of the rats was dynamically evaluated by echocardiography. Upon termination, the hearts were excised, and the infarct size of the hearts was determined by TTC staining. (A) Representative echocardiogram. (B) Representative photograph of infarct size. (C) Statistical analysis of infarct size. Data are presented as mean ± SD. *n* = 6. ^###^*p* < 0.001 vs. sham group. ***p < 0.001 vs. HF group.

Permanent ligation of the left coronary artery caused damage to cardiac function damage, and the values of EF and FS were significantly lower than those in the sham group (36.26 ± 4.14% vs. 95.23 ± 2.80% for EF; 17.88 ± 1.32% vs. 75.05 ± 3.70% for FS; *p* < 0.001; Table 1, Figure 2(A)). ASI improved cardiac function with significantly increased EF and FS (75.27 ± 5.75% vs. 36.26 ± 4.14% for EF; 45.39 ± 3.66% vs. 17.88 ± 1.32% for FS; *p* < 0.001 vs. HF group). In addition, impaired cardiac function resulted in enlargement of the cardiac chamber and increase of LVIDd and LVIDs (9.32 ± 0.50 mm vs. 5.97 ± 0.37 mm for LVIDd; 7.56 ± 0.43 mm vs. 1.53 ± 0.26 mm for LVIDs; *p* < 0.001 vs. sham group). ASI significantly reduced the two values (7.49 ± 0.62 mm vs. 9.32 ± 0.50 mm for LVIDd; 4.41 ± 0.49 vs. 7.56 ± 0.43 mm for LVIDs; *p* < 0.001 vs. HF group).

**Table 1.** Cardiac function of the rats evaluated by echocardiography.

Parameters	Sham	HF	HF + ASI
EF (%)	95.23 ± 2.80	36.26 ± 4.14^###^	75.27 ± 5.75***
FS (%)	75.05 ± 3.70	17.88 ± 1.32^###^	45.39 ± 3.66***
LVIDd (mm)	5.97 ± 0.37	9.32 ± 0.50^###^	7.49 ± 0.62***
LVIDs (mm)	1.53 ± 0.26	7.56 ± 0.43^###^	4.41 ± 0.49***

Data are presented as mean ± standard deviation. *n* = 6 for each group. ^###^*p* < 0.001 vs. sham group. ***p < 0.001 vs. HF group.

### ASI reduced infarct size

Permanent ligation of the left coronary artery resulted in the ischaemia of the left ventricular myocardium followed by severe infarction (Figure 2(B)). Compared with the rats in the HF group, ASI significantly reduced the infarct size of the left ventricle (20.69 ± 2.98% vs. 39.11 ± 3.97%; *p* < 0.001; Figure 2(C)).

### ASI protected against oxidative stress

**Figure 3.**
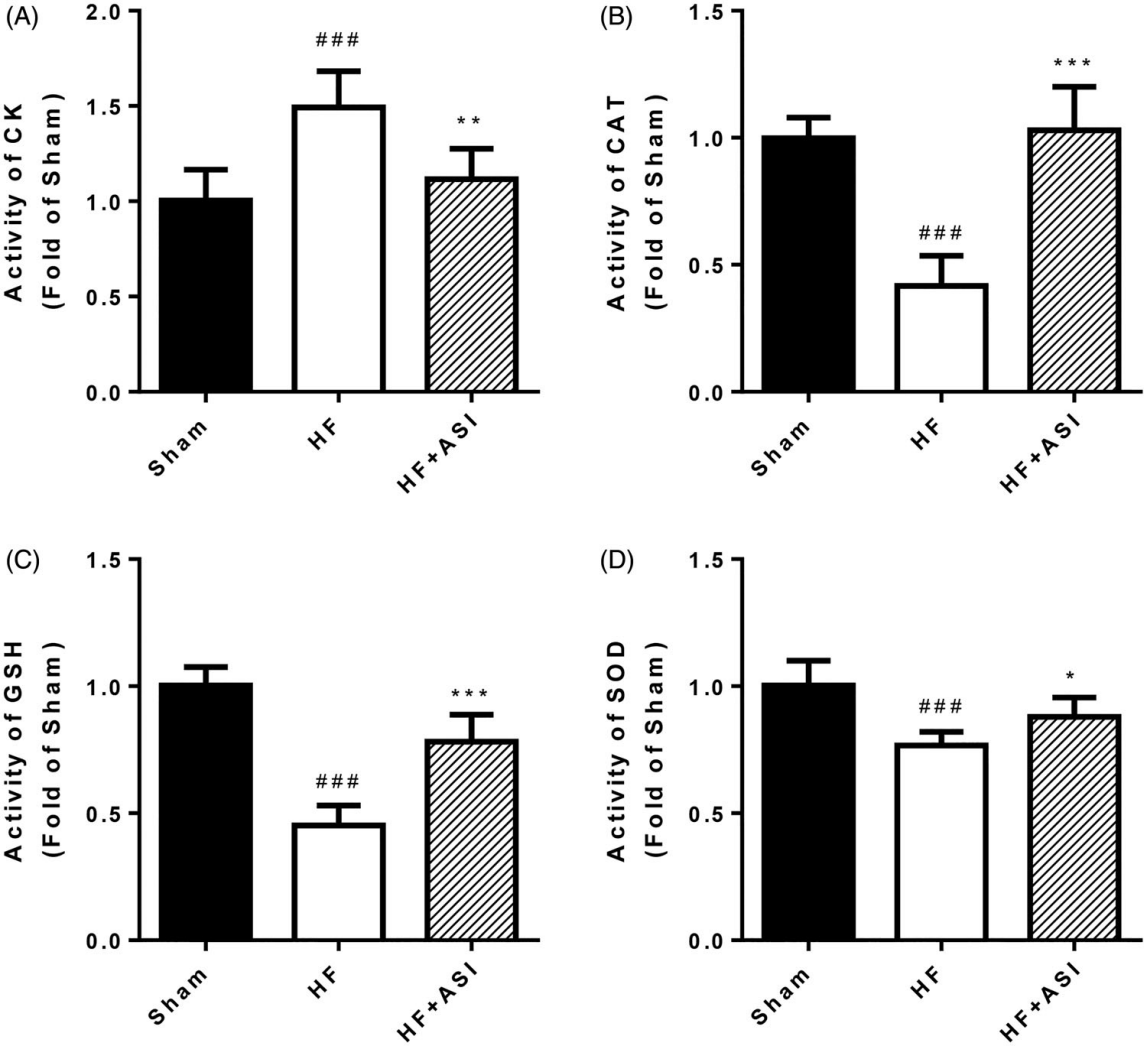
Oxidative stress in the ventricular myocardium. Upon termination, the levels of creatine kinase (A), catalase (B), glutathione (C) and superoxide dismutase (D) in the border zone of infarcted ventricular myocardium are determined. Data are presented as mean ± SD. *n* = 6. ^###^*p* < 0.001 vs. sham group. ***p < 0.001 vs. HF group. **p < 0.01 vs. HF group. *p < 0.05 vs. HF group.

The significant (*p* < 0.001) decrease of antioxidant defense molecules (such as CAT, GSH and SOD) in the myocardium of HF rats led to the leakage of CK from the damaged cardiomyocytes (Figure 3). ASI effectively maintained the levels of CAT (*p* < 0.001), GSH (*p* < 0.001) and SOD (*p* < 0.05) and prevented the leakage of CK (*p* < 0.01).

### Nrf2/HO-1 pathway was involved in the antioxidant effect of ASI

**Figure 4.**
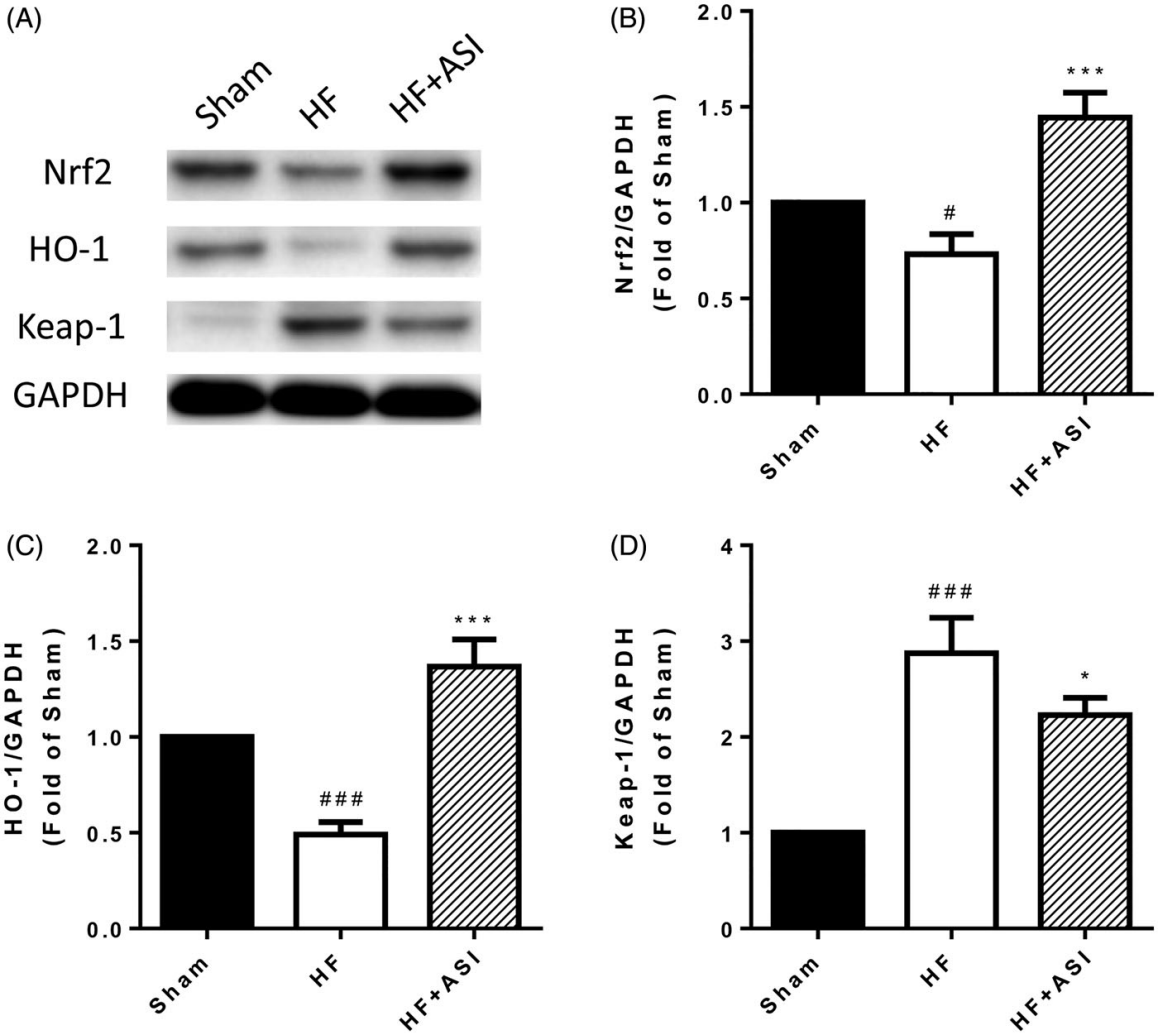
Nrf2/HO-1 pathway. The protein expression of Nrf2, HO-1 and Keap-1 in the ventricular tissue are determined by Western blot. (A) Representative photographs of Western blot. (B–D) Statistical analysis of Western blot. Data are presented as mean ± SD. ^###^*p* < 0.001 vs. sham group. ^#^*p* < 0.05 vs. sham group. ***p < 0.001 vs. HF group. *p < 0.05 vs. HF group. Experiments were repeated at least three times.

Western blot showed that the protein expression of Nrf2 and HO-1 were decreased in the myocardial tissue of HF rats, while ASI significantly increased the protein expression of Nrf2 and HO-1 by 1.97-fold and 2.79-fold, respectively (Figure 4). In contrast, the protein expression of Kelch-like ECH-associated protein 1 (Keap-1), a negative regulator of Nrf2, was elevated after the occurrence of heart failure but reduced after ASI treatment (Figure 4).

### ASI induced the nuclear translocation of Nrf2, followed by transcriptional activation

**Figure 5.**
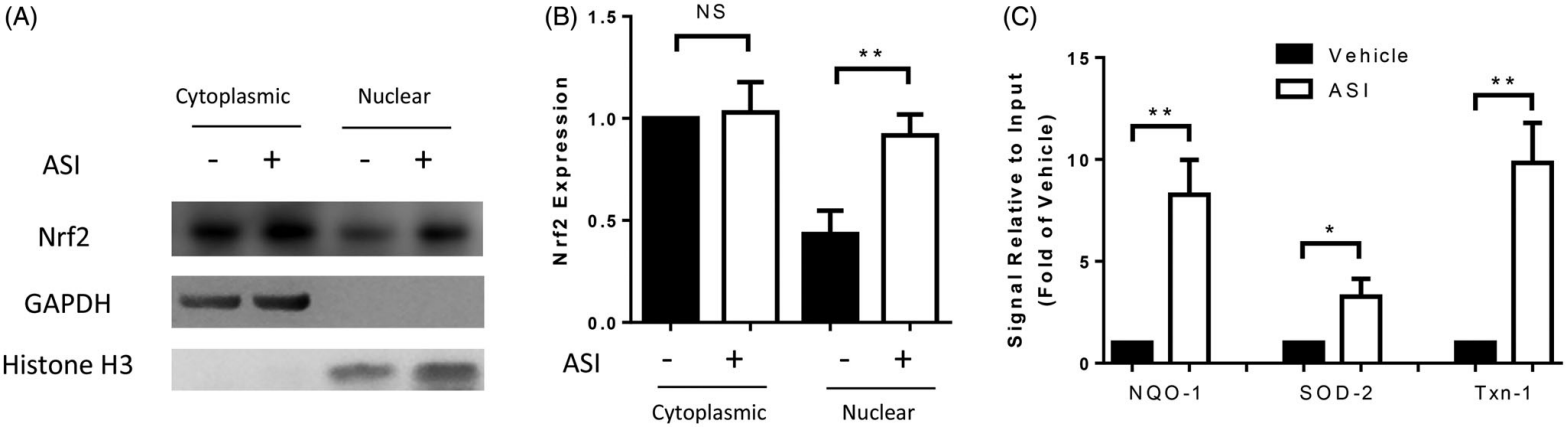
Transcription activated by Nrf2. The entire nuclear content was isolated from the H9c2 cells to perform Western blot (A, B) or chromatin immunoprecipitation with a Nrf2 antibody (C). The transcription of the downstream genes was analyzed by real-time PCR. Data are presented as mean ± SD. **p < 0.01. *p < 0.05. NS: no significance. Experiments were repeated at least three times.

By isolating the cytoplasmic and nuclear proteins from the H9c2 cells, we observed a significantly increased level of nuclear Nrf2 protein after ASI treatment, but the cytoplasmic Nrf2 protein level was unchanged (Figure 5(A,B)). To investigate the underlying mechanism by which Nrf2 activated downstream gene promoters, we performed ChIP with isolated nuclear extracts. As a result, the promoters of NQO-1, SOD-2 and Txn-1 were significantly activated (8.27, 3.27 and 9.83-fold, respectively) after ASI treatment (Figure 5(C)).

## Discussion

There were considerable pieces of evidence showing that ASI improved cardiac function in rat and mouse models of heart failure which was induced by left coronary artery ligation (Zhao et al. 2009; Wang et al. 2010; Dong et al. 2017). Consistent with the previous studies, our present work showed the protective effects of ASI on cardiac function through elevating EF and FS and reducing LVID. According to most of the published reports, the EF values of SD rats should be in an approximate range between 60 and 85%. In our study, we recorded relatively high EF values of up to 95%. The possible reasons might be the calibration of the instrument, location and position of the probe, and the remaining hair after shaving off. In addition, we observed a significant reduction in the infarct size of the left ventricle in HF rats with ASI treatment. ASI was reported to counteract oxidative stress in both *in vitro* and *in vivo* studies (He et al. 2013; Li et al. 2013). However, whether antioxidation was involved in the protection from heart failure with ASI was still not known. In this study, we reported that ASI is effective in maintaining the levels of CAT, GSH and SOD and preventing the leakage of CK, which suggested that ASI protected against heart failure partly by alleviating oxidative stress.

Nrf2 is a key regulator of redox homeostasis and cellular antioxidant defences. It has been reported *in vitro* that Nrf2 plays a critical role in the protective effect of ASI against ischaemia/reperfusion damages in cortical neurons (Gu et al. 2015), and against LPS-induced inflammation in microglial cells (Li C et al. 2018). Nrf2 is also known for improving blood–brain barrier integrity from LPS-induced disruption in mice (Li H et al. 2018). In this study, we reported increased protein expression of Nrf2 and its downstream signal molecule HO-1 in the myocardial tissue of HF rats. Such a result was consistent with recently published data showing that ASI induced Nrf2/HO-1 protein expressions in the myocardium in a rat model of abdominal aortic constriction (Nie et al. 2019).

Under homeostatic conditions, Nrf2 is sequestered in the cytoplasm by Keap-1 which interacts with Nrf2 in a redox-sensitive manner. The dissociation of the proteins in the cytoplasm is followed by the transportation of Nrf2 to the nucleus to induce transcription (Kang et al. 2005). In our current work, we reported a decreased level of Keap-1 protein along with increased translocation of Nrf2 from the cytoplasm to the nucleus. In the nucleus, Nrf2 functions as a transcription factor by binding to a cis-acting enhancer sequence known as ARE to regulate the transcription of antioxidant genes and the other cytoprotective phase II detoxifying enzymes, such as HO-1, SOD, GPx, GST, NQO-1, Txn-1, and Txnrd-1 (Li et al. 2010). Intriguingly, according to our ChIP analysis, the promoters of NQO-1, SOD-2 and Txn-1 were significantly activated after ASI treatment, indicating that their activation might be directly responsible for the antioxidant effect of ASI.

Although we provided the first evidence that ASI is capable of inducing the downstream signalling pathways of Nrf2, it is still unknown whether other factors can induce the Nrf2 signalling. Previous studies have shown that hyperoxia can stimulate the Nrf2-ARE transcriptional response via ROS-EGFR-PI3K-Akt/ERK MAPK signalling in pulmonary epithelial cells (Papaiahgari et al. 2006). In cortical neurons, ASI-induced Nrf2 activation was mediated by EGFR and protected against ischaemia/reperfusion damage *in vitro* (Gu et al. 2015). ASI exerted anti-neroinflammatory effects in LPS-induced microglial cells by inducing the Nrf2/HO-1 activation via inhibiting the ERK pathway (Li C et al. 2018). All these findings prompt us to investigate the possible role of the EGFR/ERK pathway in mediating the protective effects of ASI against heart failure in our future work.

## Conclusions

Using a rat model of left coronary artery ligation, we first proved that ASI protects rats from heart failure by counteracting oxidative stress. The experiment disclosed the role of Nrf2 in mediating the antioxidative properties of ASI. We further highlighted the function of ASI in promoting the translocation of Nrf2 from cytoplasm to the nucleus to induce the transcription of downstream antioxidant genes. Further investigation is warranted before the application of ASI in clinical practice.
